# Volatile Organic Compounds Produced by *Kosakonia cowanii* Cp1 Isolated from the Seeds of *Capsicum pubescens* R & P Possess Antifungal Activity

**DOI:** 10.3390/microorganisms11102491

**Published:** 2023-10-04

**Authors:** José Luis Hernández Flores, Yomaiko Javier Martínez, Miguel Ángel Ramos López, Carlos Saldaña Gutierrez, Aldo Amaro Reyes, Mariem Monserrat Armendariz Rosales, Maraly Jazmin Cortés Pérez, Mayela Fosado Mendoza, Joanna Ramírez Ramírez, Grecia Ramírez Zavala, Paola Lizeth Tovar Becerra, Laila Valdez Santoyo, Karen Villasana Rodríguez, José Alberto Rodríguez Morales, Juan Campos Guillén

**Affiliations:** 1 Centro de Investigación y de Estudios Avanzados del IPN, Irapuato 6824, Mexico; jose.hernandezf@cinvestav.com.mx; 2 Facultad de Química, Universidad Autónoma de Querétaro, Cerro de las Campanas S/N, Querétaro 76010, Mexico; yomaiko_javier@outlook.com (Y.J.M.); agromyke@gmail.com (M.Á.R.L.); aldo.amaro@uaq.edu.mx (A.A.R.); mararmendarizr@gmail.com (M.M.A.R.); mcortes15@alumnos.uaq.mx (M.J.C.P.); mfosado10@alumnos.uaq.mx (M.F.M.); jramirez175@alumnos.uaq.mx (J.R.R.); gramirez52@alumnos.uaq.mx (G.R.Z.); ptovar29@alumnos.uaq.mx (P.L.T.B.); lvaldez24@alumnos.uaq.mx (L.V.S.); kvillasana07@alumos.uaq.mx (K.V.R.); 3 Facultad de Ciencias Naturales, Universidad Autónoma de Querétaro, Av. de las Ciencias S/N, Querétaro 76220, Mexico; carlos.saldana@uaq.mx; 4 Facultad de Ingeniería, Universidad Autónoma de Querétaro, Cerro de las Campanas S/N, Querétaro 76010, Mexico

**Keywords:** *Enterobacteriaceae*, 2-butanone, 3-hydroxy, *Capsicum pubescens* seeds, fungal inhibition, biocontrol, genome sequencing

## Abstract

The *Kosakonia cowanii* Cp1 strain was isolated from seeds of *Capsicum pubescens* R. & P. cultivated in Michoacan, Mexico. Genetic and ecological role analyses were conducted for better characterization. The results show that genome has a length of 4.7 Mbp with 56.22% G + C and an IncF plasmid of 128 Kbp with 52.51% G + C. Furthermore, pathogenicity test revealed nonpathogenic traits confirmed by the absence of specific virulence-related genes. Interestingly, when fungal inhibitory essays were carried out, the bacterial synthesis of volatile organic compounds (VOCs) with antifungal activity showed that *Sclerotinia sp*. and *Rhizoctonia solani* were inhibited by 87.45% and 77.24%, respectively. Meanwhile, *Sclerotium rolfsii*, *Alternaria alternata*, and *Colletotrichum gloeosporioides* demonstrated a mean radial growth inhibition of 52.79%, 40.82%, and 55.40%, respectively. The lowest inhibition was by *Fusarium oxysporum,* with 10.64%. The VOCs’ characterization by headspace solid–phase microextraction combined with gas chromatography–mass spectrometry (HS-SPME-GC–MS) revealed 65 potential compounds. Some of the compounds identified with high relative abundance were ketones (22.47%), represented by 2-butanone, 3-hydroxy (13.52%), and alcohols (23.5%), represented by ethanol (5.56%) and 1-butanol-3-methyl (4.83%). Our findings revealed, for the first time, that *K. cowanii* Cp1 associated with *C. pubescens* seeds possesses potential traits indicating that it could serve as an effective biocontrol.

## 1. Introduction

Pepper (*Capsicum* spp.) has an exciting history of domestication and has seen more than 6000 years of use in diverse foodstuffs in Mexico, such as in fresh, dried, or processed products. It is believed that this crop originated in South America [1,2,3]. Therefore, Mexico and Central America are considered genetic diversity hotspots that have generated important domesticated varieties of pepper. These peppers have relevance in the agriculture and food industry, specifically those industries that are linked to the use of pigments, shape, size, appearance, flavor, pungency, and nutritive components [4,5]. In addition, historical records indicate that *Capsicum* was introduced to Spain by Christopher Columbus after discovering America in 1493, and then to the rest of Europe and—eventually—to India, Asia, and Africa [6]. Geographical displacement and domestication processes such as artificial and natural selection in agricultural environments have led to the existence of a great number of species in the *Capsicum* genus. However, there are five important cultivated species of economical relevance that are considered here: *C. annuum* L., *C. frutescens* L., *C. chinense* Jacq., *C. baccatum* L., and *C. pubescens* R. & P. [1,2,3,4,5,6].

Therefore, all agronomic management approaches for pepper cultivation during historical domestication are important to consider in terms of obtaining fruits and seeds with high quality for diverse purposes [3]. From these significant traits, genetic lines with resistances to diverse pathogens, such as viruses, bacteria, and fungi, are of ecological and economical relevance during plant and seed development stages [7,8,9,10]. In addition to modern breeding programs, recently, diverse methodologies, such as metagenomics, transcriptomics, proteomics, or metabolomics approaches, have been relevant to understanding microorganism–plant interactions or plant development during the lifecycle of *Capsicum* [11,12,13,14,15].

Throughout the last decade, the study of microbiomes on the seeds from diverse plant species, including *Capsicum*, has increased due to its ecological importance [16,17,18]. These studies highlight the metabolic activities of microorganism endophytes or epiphytes on seed germination, seedling establishment, plant growth, and plant development stages and as a biocontrol [16,17,18]. Moreover, it is of special interest that the variations in the diversity of seed-associated microorganisms (SAMs) could be determined by their plant genotype as well as by environmental and soil management practices [16]. Therefore, the characterization of the SAMs on *Capsicum* with beneficial or detrimental traits is necessary during agricultural practices for an assessment of seed quality and plant development.

Although the origin or transmission route of SAMs is under study, it is interesting to understand the recruitment of diverse communities of microorganisms during plant seed development and to evaluate their metabolic potential through molecule production and characterization, which may indicate ecological significance and agricultural applications [16]. Thus, for instance (and of particular interest for our research work), the diverse members of the family *Enterobacteriaceae* were isolated from seeds of a xerophytic plant, *Lactuca serriola*. Among them is *Kosakonia cowanii*, which possesses the ability to produce a high concentration of exopolysaccharides with effects on plant drought tolerance [19]. In addition, *K. cowanii* has been isolated from diverse crops with plant-promoting activities through auxin (IAA) and siderophore production [20,21]. Also, the great metabolic ability of the *Kosakonia* genus has been characterized by the diverse strains isolated from insects, plants, human, or other sources [22,23,24,25,26,27,28,29,30]. Recently, our research group reported the presence of *K. cowanii* in chili powder via 16s rRNA library sequencing [31], and the isolation of a *K. cowanii* Ch1 strain with the ability to produce active VOCs against certain fungal pathogens and colonize the seeds of *C. annuum* L. [32] was also achieved. These results allow us to hypothesize that *K. cowanii* could be associated with fruits or seeds of *C. annuum* L.

Although systematic research of SAMs in *Capsicum* is limited, this plant is cultivated around the world, and its production is close to 34.5 million tons annually. Furthermore, in Mexico, it is one of the most important economic and agricultural crops, cultivated on a total of 150 million hectares [33]. Therefore, the high diversity of genetic varieties of *Capsicum* plants that are grown in diverse soils and environmental conditions make it necessary to explore SAMs as a source of beneficial microorganisms. In the present report, we hypothesize that *K. cowanii* could be associated with *Capsicum* seeds. Therefore, our results show, for the first time, that the presence of *K. cowanii*, specifically, the strain associated with *Capsicum pubescens* seeds, has an important ecological role in terms of controlling pathogenic fungi. In addition, this opens the possibility of a further exploration of the SAMs in the *Capsicum* that are cultivated under different environmental conditions in Mexico.

## 2. Materials and Methods

### 2.1. Bacterial Strain Isolation

Serrano pepper (*Capsicum annuum* L.) and manzano pepper (*Capsicum pubescens*) fruits without visual damage or infections were obtained from cultivated plants in Querétaro and Michoacán, México. Then, they were surface disinfected with sodium hypochlorite (10% *v*/*v*) for 10 min; this was followed by disinfection with ethanol (50% *v*/*v*) for 10 min. Then, they were air dried at room temperature in a biosafety cabinet. The seeds were placed on tryptic soy agar (TSA) medium (Difco Laboratories, Detroit, MI, USA) with 100 μg/mL of ampicillin and incubated at 37 °C for 24 h. Bacterial colonies were isolated in the same culture medium. For taxonomy identification, the 16s rDNA gene was amplified by PCR, and amplicon was sequenced at Macrogen Inc. (Seoul, Republic of Korea). The sequences obtained were analyzed by neighbor-joining method in MEGA X software and compared with the sequences of *K. cowanii* obtained from NCBI database [34,35,36,37].

### 2.2. Pathogenicity Test

A pathogenicity test was conducted using five bacterial strains isolated from *C. pubescens* seeds (which were identified as *K. cowanii*), following the methodology in our previous report [32]. In general, seeds of *Capsicum annuum* L. var. serrano were disinfected in sodium hypochlorite (10%, *v*/*v*) for 5 min and rinsed with sterile distilled water. Germination was carried out in seedling trays under greenhouse conditions. After 45 to 50 days post-germination, seedlings were transplanted into pots. Plants with 13–14 true leaves were used for pathogenicity test. On the other hand, fruits without damage or infection were disinfected with sodium hypochlorite (10%, *v*/*v*) for 10 min, ethanol (50%, *v*/*v*) for 10 min, and, finally, rinsed with sterile distilled water. Bacterial suspension (10 μL) with approximately 1 × 10^8^ CFU/mL was inoculated on six leaves of each plant in triplicate and on three pepper fruits; both were damaged with a sterile needle and inoculated at that zone. Negative controls were inoculated with sterile distilled water. Inoculated plants were kept in greenhouse conditions while inoculated fruits were placed inside of sealed container at room temperature. The plants were observed for 5–7 days.

### 2.3. Genome Sequencing and Assembly

We randomly selected the *K. cowanii* strain Cp1 based on pathogenicity test and fungal inhibitory essays to isolate its genomic DNA for sequencing. DNA was extracted with the ZymoBIOMICS^TM^ DNA Miniprep Kit (Zymo Research, Irvine, CA, USA) according to the manufacturer’s instructions. We used the Genomic Sequencing Service at Zymo Research, Irvine, CA, USA, which used the NovaSeq^®^ (Illumina, San Diego, CA, USA) platform. Sequence reads were processed at the Bacterial and Viral Bioinformatics Resource Center (BV-BRC). First, a Fastq was used to filtrate the sequences [38]. Second, for genome annotation, the RAST tool kit (RASTtk) was used [39]. Lastly, the circular genome map was modeled in CIRCOS software [40]. The genome statistics were as follows: coarse consistency (98.7%), fine consistency (98%), CheckM completeness (99.7%), and CheckM contamination (0.1%). The bacterial phylogenetic tree was constructed by using the pipelines at BV-BRC, where Mash/MinHash [41], PGFams [38], MUSCLE v5 [37], RaxML v8.2.11 [37], and fast bootstrapping [38] were included in order to conduct the phylogenetic analysis. PlasmidSPAdes version v3.13.0, with default settings, was used for the plasmid assembly [42]. DNA sequences were deposited in NCBI as BioProject ID PRJNA1003013. The genome accession number is JAUZWC000000000.

### 2.4. Antibiotic Susceptibility Testing

The antimicrobial phenotype of *K. cowanii* Cp1 and *Escherichia coli* XL1 blue as a control were evaluated using the guidelines of CLSI [43]. In general, bacterial strains were inoculated in tryptic soy broth (TSB) medium and growth at 37 °C and shaken until an optical density (OD) between 0.4 and 0.5 at 600 nm was achieved. Then, 100 μL of bacterial culture was spread on Mueller–Hinton agar (Bioxon). The antimicrobial test discs (Oxoid) were as follows: penicillin (10 U), ampicillin (10 μg), carbenicillin (100 μg), dicloxacillin (1 μg), and amoxicillin/clavulanic acid (20/10 μg); cephalosporins were cephalothin (30 μg) and cefotaxime (30 μg); fluoroquinolones were ciprofloxacin (5 μg) and norfloxacin (10 μg); aminoglycosides were amikacin (30 μg), gentamicin (10 μg), and netilmicin (30 μg); macrolides were clindamycin (30 μg) and erythromycin (15 μg); carbapenems were imipenem (10 μg) and meropenem (10 μg); chloramphenicol (30 μg); nitrofurantoin (300 μg), tetracycline (30 μg); trimethoprim-sulfamethoxazole (25 μg) and the glycopeptide vancomycin (30 μg). Culture plates were incubated overnight at 37 °C and the inhibitory or clear zones around the disc were recorded in mm, according to guidelines.

### 2.5. Inhibitory Effects of VOCs

The following fungal strains were provided by the Laboratory of Plants and Agriculture Biotechnology at Queretaro University, México: *Alternaria alternata*, *Colletotrichum gloeosporioides*, *Fusarium oxysporum*, *Rhizoctonia solani*, *Sclerotium rolfsii*, and *Sclerotinia* sp. The growing conditions were as follows: potato dextrose agar (PDA) medium (Difco Laboratories, Detroit, MI, USA) kept at 28 °C over a 5- to 7-day period, conducted according to the growth rate of each fungal strain.

The inhibitory assays were evaluated by radial mycelial growth according to the methodology described previously in [32]. In general, the two-compartment plastic plate device was sealed with parafilm to avoid VOC loss. On the lower side of the device, which was used with TSA medium (Difco Laboratories, Detroit, MI, USA), a bacterial suspension (1 × 10^8^ CFU/mL) was inoculated. On the upper side of the device, which was used with PDA medium (Difco Laboratories, Detroit, MI, USA), a fungal growth disk was inoculated. Each control was tested for fungi and growth on PDA medium (Difco Laboratories, Detroit, MI, USA). Each device was an experimental unit and was incubated at 28 °C to obtain the radial mycelial growth according to the following equation: mycelial growth inhibition (%) = [(dc−dt)/dc] × 100, where dc and dt represent the mycelial growth diameters (in mm) of the control and treatment groups, respectively. The experiments were conducted in triplicate for a statistical analysis of variance and for Duncan’s multiple range test (*p* = 0.05), which were performed using DPS V12.01 software. Alternatively, bacterial antagonism toward *S. rolfsii* was tested by dual-culture assay on PDA to prove additional mechanism of inhibition. Bacterial strain (10 μL, 1 × 10^8^ CFU/mL) was inoculated close to and around fungal disk. The essay was kept at 28 °C over a 2- to 3-day period.

### 2.6. HS-SPME-GC–MS Analysis of VOCs

As the first step in obtaining a bacterial culture with volatile organic compounds (which have inhibitory effects on *Sclerotium rolfsii*), a cell-free filtrate was obtained of *K. cowanii* Cp1 grown on Tryptic soy broth (TSB) medium, with continuous shaking (100 rpm) at 37 °C, which was sampled every 6 h up to a total of 48 h. TSB medium without *K. cowanii* Cp1 strain was used as the control. The cell-free filtrate was obtained via centrifugation at 14,000 rpm for 5 min and after being filtered through a 0.2 μm membrane (Sigma-Aldrich). Then, 500 μL of the sample was placed on PDA medium (Difco Laboratories, Detroit, MI, USA), and a 7 mm mycelial disk of *Sclerotium rolfsii* was inoculated. The radial mycelial that was grown was evaluated according to the equation mentioned above. Furthermore, the sample that was obtained after 18 h of bacterial growth displayed the highest inhibitory effect; thus, it was used for VOC characterization. To recover the VOCs from the bacterial culture that was grown over 18 h on TSB medium, the sample was processed according to the methodology, and using the device, that was previously reported in [32].

Among the bacterial volatiles obtained, and based on the commercial availability (J.T., Baker, Fermont, Meyer, Sigma-Aldrich, Toluca, Mexico), seven standard compounds were evaluated individually on the inhibition of radial mycelial growth. Using the previous two-compartment plastic plate device, different amounts of individual VOCs were added to sterile filter papers on one side and on the upper side of the device, and an *S. rolfsii* growth disk of 8 mm was inoculated on PDA medium. The amounts VOCs assayed were: 50, 100, and 150 μL/plate of 2-Butanone; 50, 100, and 150 μL/plate of 2-Butanone, 3-hydroxy; 10, 20, and 40 μL/plate of 2-Nonanone; 50, 100, and 150 μL/plate of acetone; 5, 10, and 20 μL/plate of acetic acid (unidentified compound as VOC component in *K. cowanii* Cp1); 50, 100, and 150 μL/plate of benzyl alcohol; and 50, 100, and 200 μL/plate of ethanol. Sterile distilled water was used as control. Each device was incubated at 28 °C for a 3-day period to obtain the radial mycelial growth, according to equation previously described. The experiments were conducted in triplicate.

## 3. Results

### 3.1. Isolation of K. cowanii from Capsicum pubescens Seeds

**Figure 1:**
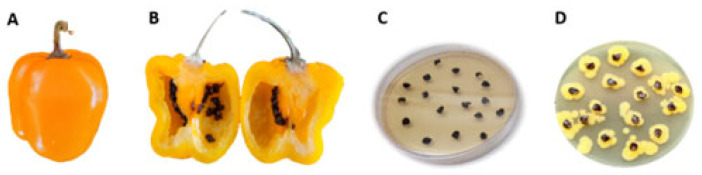
Isolation of *Kosakonia cowanii* Cp1. (**A**) *Capsicum pubescens* fruits with no infection symptoms; (**B**) interior of *Capsicum pubescens* fruits displaying seeds with no infection symptoms; (**C**) *Capsicum pubescens* seeds on TSA medium that was supplemented with 100 μg/mL of ampicillin; (**D**) a growth of *Kosakonia cowanii* after 24 h of incubation.

**Figure 2:**
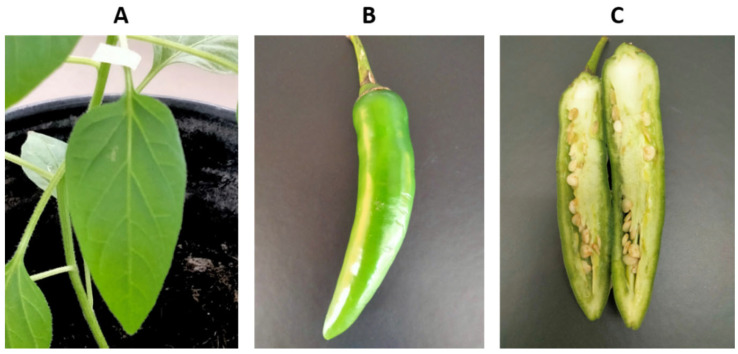
Pathogenicity test of *K. cowanii* Cp1 in a serrano pepper (*Capsicum annuum* L.). (**A**) Assay of *K. cowanii* Cp1 that grew on serrano pepper leaves; (**B**) assay of *K. cowanii* Cp1 on the external part of pepper fruits; (**C**) assay of *K. cowanii* Cp1 on the internal part of pepper fruits. No lesions were observed in any case.

Seeds from *C. annuum* and *C. pubescens* were obtained and processed for bacterial isolation on TSA medium. In a previous work [32], we isolated a *K. cowanii* strain that was resistant to β-lactam antibiotics; as such, in this case, we decided to supplement the TSA medium with 100 μg/mL of ampicillin in order to determine if this could be a similar case. From diverse seeds that were obtained from the fruits of *Capsicum* spp., a bacterial growth was noted on the seeds of manzano peppers (*C. pubescens*) that were obtained from Michoacan, Mexico. In 100% of the seeds of one fruit, there was a similar phenotype to *K. cowanii* (Figure 1). The bacterial isolates from the seeds were streaked on the same culture medium so as to obtain isolated colonies. Five bacterial colonies were selected and identified by using the 16s rDNA gene as a molecular marker. Sequence analysis confirmed them as *K. cowanii*. Then, we conducted a pathogenicity test for the *K. cowanii* strains to evaluate the phytopathogenic traits, as previously reported [32]. The infection assays on the fruits and leaves of *Capsicum annuum* L. showed no infection symptoms (Figure 2).

### 3.2. General Genome and the Plasmid Features of K. cowanii Strain Cp1

**Figure 3:**
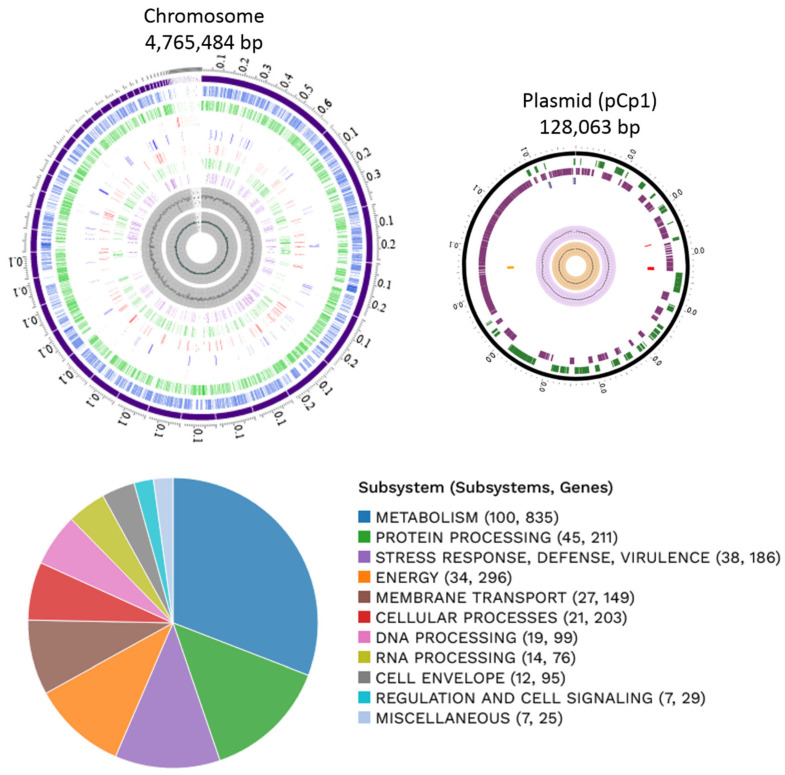
Graphical circular map of the chromosome and plasmid of *K. cowanii* Cp1. From the outside to the center rings are the ensembled contigs, CDS on forward, CDS on reverse, non-CDS, AMR genes, VF genes, transporters, drug targets, GC content, and GC skew. The subsystem functional assignments are represented in the figure below.

From these bacterial isolates, based on fungal inhibitory essays (explained below), one of them was selected randomly for genomic characterization. The genome of *K. cowanii* Cp1 has a total length of 4,765,484 bp, 56.22% of G + C, and a plasmid (pCp1) of 128,063 bp with 52.51% of G + C(Figure 3). The annotation results indicated 4509 protein coding sequences (CDS) and 89 RNA genes (84 tRNA and 5 rRNA). The genome functional subsystem categories are shown in Figure 3, where the metabolism category is represented at 37.88%; protein processing at 9.57%; the stress response, defense, and virulence category at 8.43%; energy at 13.43%; membrane transport at 6.76%; cellular processes at 9.21%; DNA processing at 4.49%; RNA processing at 3.44%; cell envelope at 4.31%; and regulation and cell signaling at 1.31%. On the pCp1 plasmid, 105 hypothetical proteins and 59 proteins with subsystem functional assignments were detected. The BLASTN analysis of plasmid pCp1 showed a 49% alignment and 94.22% similarity with the plasmid 888-76-1 identified in the *K. cowanii* JCM 10956 strain. The plasmid Wem22 identified in the *K. cowanii* SMBL-WEM22 strain showed a 46% alignment and 94.22% similarity. The plasmid unnamed1 that was identified in the phytopathogenic *K. cowanii* strain Pa82 showed a 45% alignment and 95.0% similarity. When the pCh1 plasmid identified in the *K. cowanii* Ch1 strain that was isolated from chili powder was compared with the pCp1 plasmid, the sequence coverage and similarity was 100%. The pCp1 and pCh1 are IncF plasmids that have VapBC toxin–antitoxin systems; therefore, the presence or association of IncF plasmids with the *K. cowanii* isolates obtained from chili powder and *Capsicum pubescens* could be important. As such, additional research is necessary to understand IncF plasmid’s functionality.

Additionally, according to the reference database for bacterial virulence factors (VDFB), virulence-related genes are principally classified as flagellar, iron uptake, and siderophore and vir/avr genes were not identified (Table 1).

**Figure 4:**
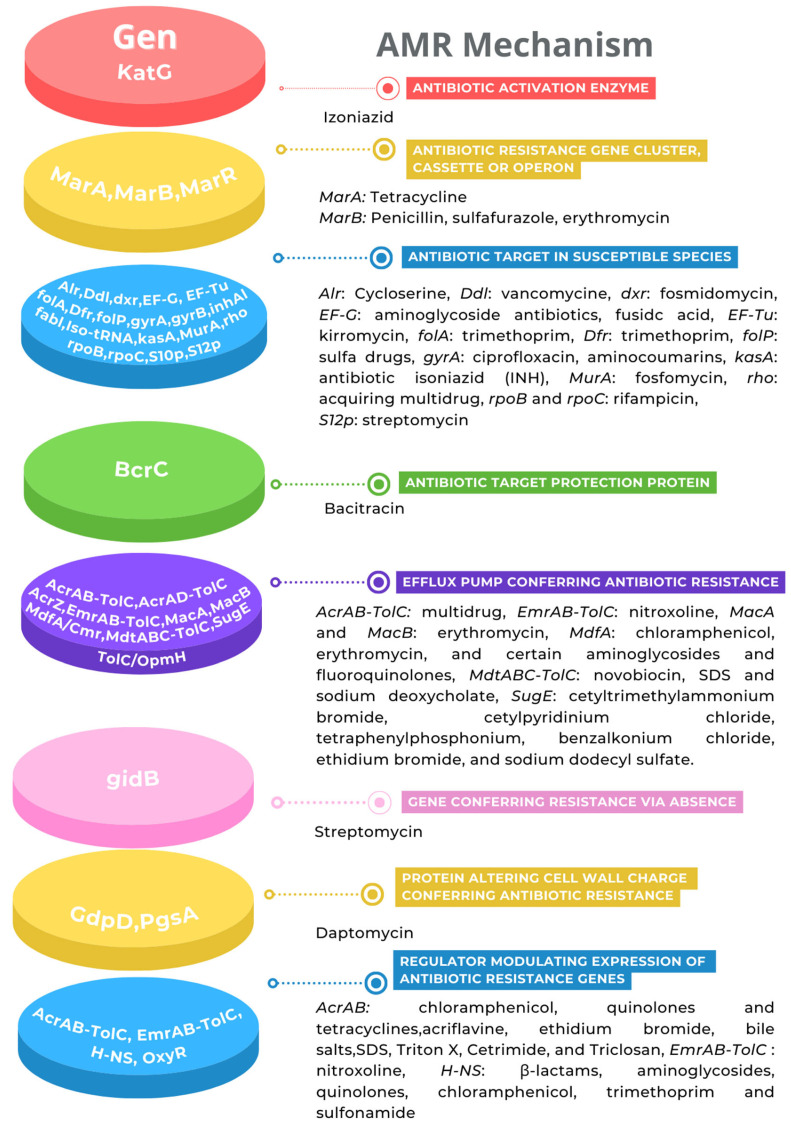
Antibiotic resistance genes. The AMR mechanism and its related genes are indicated with a color diagram.

On the other hand, the antibiotic resistance (AMR) genes predicted in the genome were classified into eight categories (Figure 4). However, the genotype must be proven with an AMR phenotype. In this regard, as a first approximation, the AMR phenotype analysis showed that *K. cowanii* Cp1 has a resistance to the penicillin antibiotic class, such as ampicillin, penicillin G, and carbenicillin, probably due to the TEM β-lactamase detected. In addition, the bacterial strain showed resistance to erythromycin, clindamycin, and vancomycin. We suspect that this is most likely due to the genes being classified as efflux pumps (Figure 4). Interestingly, the rest of the antibiotics caused growth inhibition in *K. cowanii* Cp1, supported by a clear zone of inhibition (Supplementary Figure S2), which demonstrated the importance of the phenotype versus the genotype in the analysis. Therefore, additional AMR analysis is necessary to understand the complete AMR of *K cowanii* Cp1.

**Figure 5:**
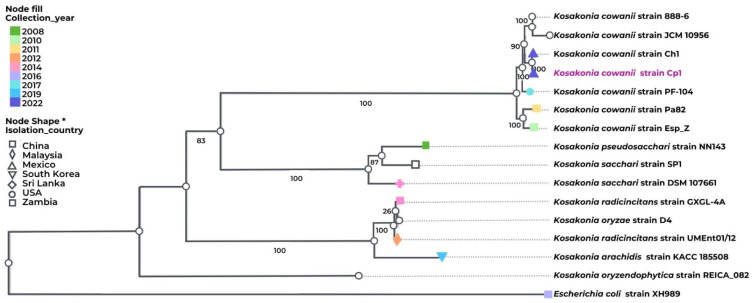
Phylogenomic analysis of *K. cowanii* Cp1. Phylogenetic analysis was performed in BV-BRC. The following strains were included: *K. cowanii strain 888-76*, *K. cowanii JCM 10956*, *K. cowanii Ch1*, *K. cowanii strain PF-104*, *K. cowanii Pa82*, *K. cowanii strain Esp Z*, *K. pseudosacchari strain NN143*, *K. sacchari SP1*, *K. sacchari strain DSM 107661*, *K. radicincitans strain GXGL-4A*, *K. oryzae strain D4*, *K. radicincitans UMEnt01/12*, *K. arachidis strain KACC 18508*, *K. oryzendophytica strain REICA 082*, and—as an outgroup—*Escherichia coli XH989*. The label color represents the collection year. The node fill and shape represent the country from which the isolates were gathered, including Mexico, China, Malaysia, Republic of Korea, Sri Lanka, USA, and Zambia.

Based on the previous genome sequences of the *Kosakonia* genus isolates from diverse sources and geographical regions, we decided to perform a phylogenomic analysis that was based on 100 core genes for *K. cowanii* Cp1 and on the related strains of the *Kosakonia* genus (Figure 5). The results showed that *K. cowanii* Cp1 is clustered with *K. cowanii strain 888-76*, *K. cowanii JCM 10956*, *K. cowanii Ch1*, *K. cowanii strain PF-104*, *K. cowanii Pa82*, and *K. cowanii strain Esp Z.* Although it is closely related to *K. cowanii* Ch1—an isolate from Mexican chili powder—the high genetic similarity between both strains means that there is strong evidence to suspect that the *K cowanii* detected in chili powder is due to its spread through *Capsicum* seeds.

### 3.3. Effects of VOCs on Mycelial Growth

**Figure 6:**
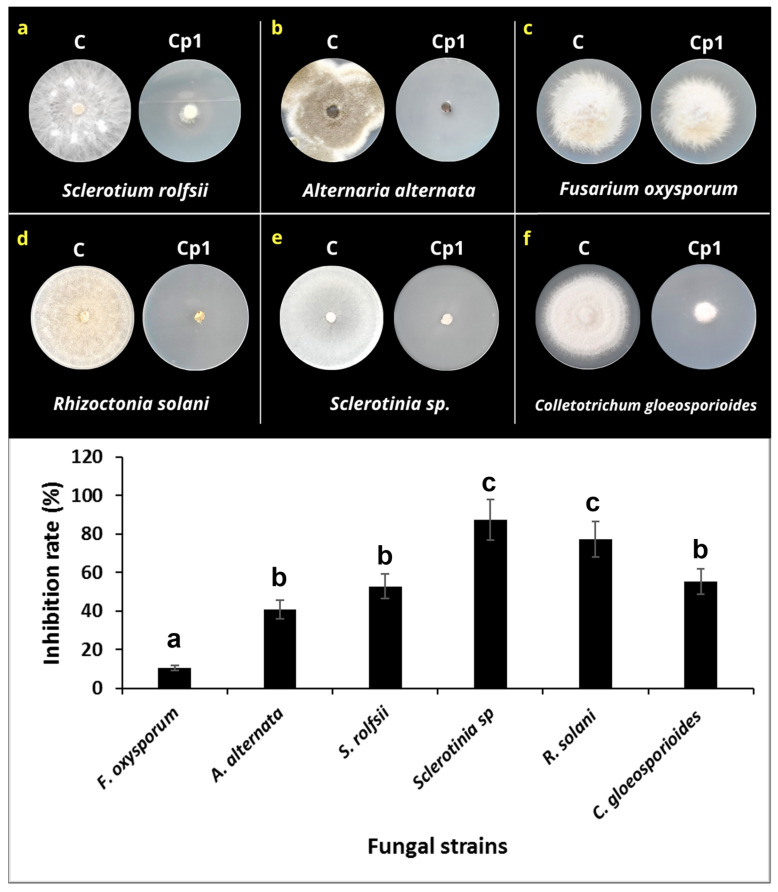
Effect of the VOCs produced by *K. cowanii* Cp1 on mycelial growth. (**a**–**f**) show the radial mycelial growth for the untreated controls (indicated by C) and those that were VOC-treated (indicated by Cp1). The lower graph shows the inhibition mycelial rate (%). Statistical differences are represented by letters.

To investigate the effect of the VOCs on mycelial growth, each one of the fungi was exposed to *K. cowanii* Cp1 in a dual-plate culture. The results in Figure 6 show that the VOCs produced by *K. cowanii* Cp1 during growth on TSA medium had a significant inhibitory effect on the mycelial growth of the tested fungi. The colony diameter of the six-fungus plates that were treated with *K. cowanii* Cp1 VOCs were significantly lower than the diameter of those grown on the control plates (Figure 6). Thus, for instance, the highest inhibition rate caused by the VOCs of all the tested fungi was observed in *Sclerotinia* sp., which exhibited an 87.45% inhibition, followed by *R. solani*, with a 77.24% inhibition. *S. rolfsii*, *A. alternata*, and *C. gloeosporioides* demonstrated a mean radial growth inhibition of 52.79%, 40.82%, and 55.40%, respectively. The lowest inhibition percentage demonstrated was by *F. oxysporum*, with a 10.64% inhibition. Alternatively, to prove additional mechanisms on fungal inhibition, a dual-culture assay on PDA was tested. At first approximation, *S. rolfsii* demonstrated a bacterial strain; however, mycelial growth was not affected (Supplementary Figure S1), suggesting that VOCs produced during fermentation could be the principal mechanism of fungal inhibition. Based on this result, we decided to analyze the VOCs’ composition.

### 3.4. Characterization of VOCs

**Figure 7:**
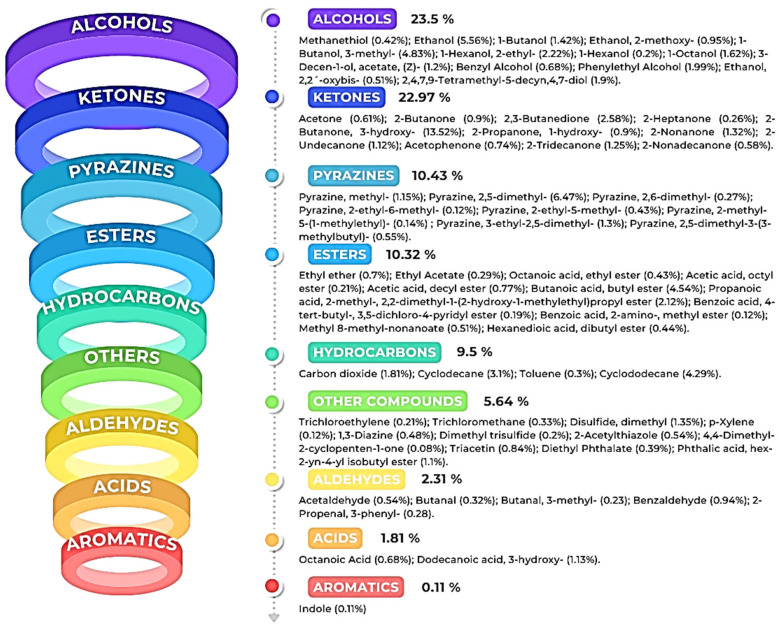
The VOCs detected in *K. cowanii* Cp1. Each figure color shows the class family of the compounds and their percentage according to their relative peak-area profile.

Characterization of the VOCs produced by *K. cowanii* Cp1 can clarify the diverse routes of metabolism that are used by this bacterial strain. With this in mind, we decided to analyze the VOCs by HS-SPME-GC–MS, which was achieved by using the bacterial culture that had 18 h of growth and was based on a 50% inhibition rate in a cell-free filtrate against *Sclerotium rolfsii*. The volatile organic compounds produced by *K. cowanii* Cp1 were compared with the control, and the results showed 65 VOCs (Table 2). They were then classified in the following functional groups (according to their total relative peak area (Figure 7)): alcohols (23.5%), ketones (22.97%), pyrazines (10.43%), esters (10.32%), hydrocarbons (9.5%), aldehydes (2.31%), acids (1.81%), and aromatics (0.11%).

**Figure 8:**
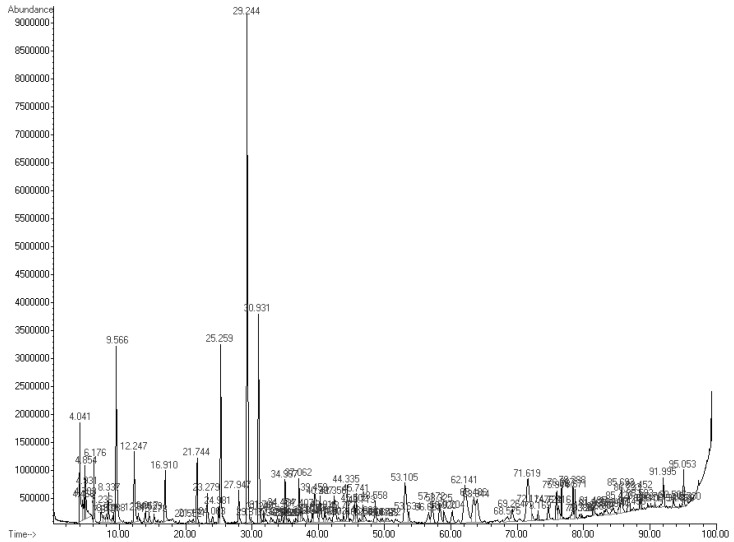
The VOC profiles of *K. cowanii* Cp1. The relative peak abundance and retention time are shown.

As is indicated in Figure 8, the six compounds with the highest relative peak-area percentages were 2-butanone, 3-hydroxy (13.52%); pyrazine-2,5-dimethyl (6.47%); ethanol (5.56%); 1-butanol-3-methyl (4.83%); butanoic acid and butyl ester (4.54%); and cyclododecane (4.29%). All of the results show that, during the growth of *K. cowanii* Cp1 on TSA medium, there was a fermentation process that produced a mixture of VOCs—some of them, most likely, exerted effects on mycelial growth inhibition. However, further analysis is necessary to understand the potential mechanisms leading to the production of each VOC and its effects on cellular processes.

### 3.5. Evaluation of Standard VOCs

**Figure 9:**
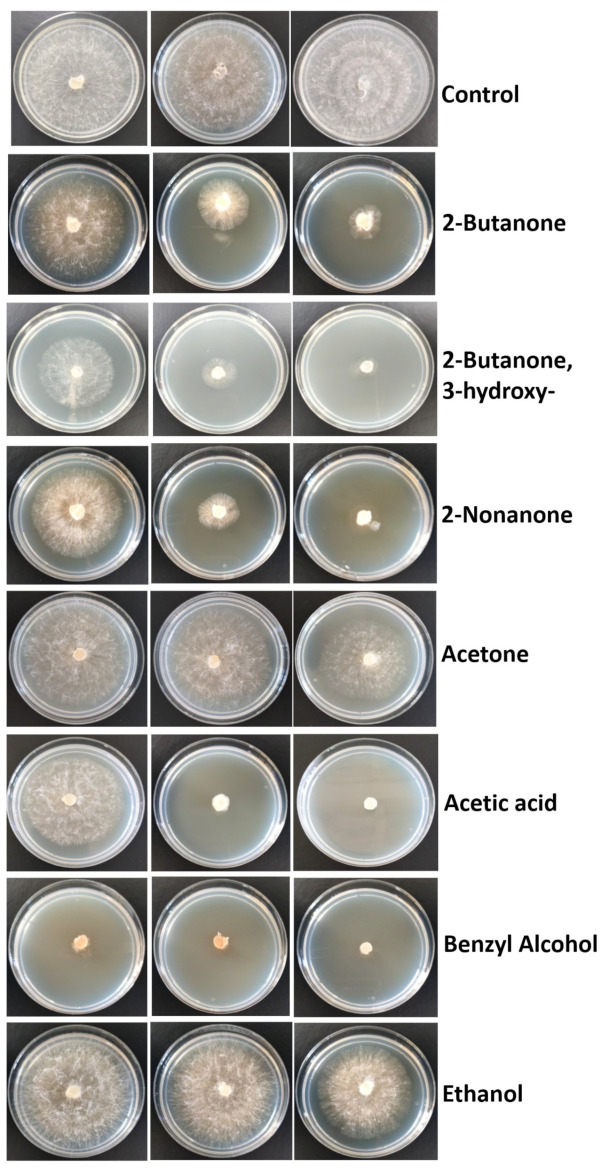
Inhibitory effect of standard VOCs on the mycelia growth of *S. rolfsii*. The amount for each compound assayed were, from left to right: 50, 100, and 150 μL/plate of 2-Butanone; 50, 100, and 150 μL/plate of 2-Butanone, 3-hydroxy; 10, 20, and 40 μL/plate of 2-Nonanone; 50, 100, and 150 μL/plate of acetone; 5, 10, and 20 μL/plate of acetic acid; 50, 100, and 150 μL/plate of benzyl alcohol; and 50, 100, and 200 μL/plate of ethanol. Sterile distilled water was used in the control.

According to the identified VOCs, we decided to analyze some compounds. The mycelial inhibition rate of the seven compounds were as follows: the strongest antifungal effect against *S. rolfsii* was benzyl alcohol, with 82.5% using 50 μL/plate and 100% using 100 and 150 μL/plate. Then, acetic acid (unidentified compound as VOC component in *K. cowanii* Cp1 and used as control positive) showed 85 and 100% inhibition using 10 and 20 μL/plate, respectively, and only 21% inhibition using 5 μL/plate. For 2-nonanone, a low inhibition of 25% was observed at 10 μL/plate, while an inhibition of 72.5 and 90% was observed with 20 and 40 μL/plate, respectively. With 2-butanone, 3-hydroxy, an inhibition of 70 and 82.5% was observed with 100 and 150 μL/plate, respectively, while the inhibition observed with 50 μL/plate was only 37.5%. With 2-butanone, inhibition was 15, 56.2, and 72.5% using 50, 100, and 150 μL/plate, respectively. Acetone only showed 25% inhibition using the highest volume (150 μL/plate). Ethanol was the compound that showed the lowest inhibition rate; only at the highest volume of 200 μL/plate was 21.25% inhibition observed (Figure 9).

## 4. Discussion

According to the hypothesis established in this research work, the findings reported here present, for the first time, the association of the *K. cowanii* strain Cp1 with seeds of *C. pubescens*, a plant that is cultivated in different agroecological zones in Michoacan, Mexico [44]. Although *C. annuum* L. seeds from Queretaro, Mexico, were analyzed, bacterial strains related to the *Kosakonia* genus were not detected; however, several other isolated bacterial genera are under study in our laboratory. Although we analyzed a relatively limited number of samples of *Capsicum* seeds, it is necessary to continue exploring in order to understand the seed association of *K. cowanii* with the diverse species of the *Capsicum* genus in the diverse geographical regions of Mexico so as to determine if the plant genotype, plant species, environment, and soil management practices have an effect on the presence of this bacterial species. This should be performed because previous results have detected the presence of *K. cowanii* in diverse samples of chili powder [16,17,18,31].

Throughout the last decade, *K. cowanii* has been isolated and associated with the microbiome of diverse organisms and processed products. Its huge metabolic versatility has shown an extensive resistance to diverse environmental factors, such as pH, salinity, temperature, and, most likely, host biomolecules, which could promote diverse bacterial interactions with diverse organisms such as insects, plants, and humans [20,21,22,23,24,25,26,27,28,29,30,31,32]. In this sense, it is widely recognized that the fruits and seeds of *C. pubescens* are a source of capsaicinoids and phenolic compounds [45]. Research works have demonstrated their antimicrobial properties as a consequence of structural or functional disruptions to the bacterial cell membrane [46]. The fact that *K. cowanii* has been isolated from *C. pubescens* seeds demonstrates that it possesses mechanisms of resistance to these compounds. In this sense, the multidrug efflux pumps of MdtABC-TolC that were detected in *K. cowanii* Cp1 have been characterized as important components of *Salmonella* sp. during colonization in the intestine and of virulence in mice [47]. In addition, this multidrug efflux pump in the phytopathogenic *Erwinia amylovora* plays an important role during colonization and resistance to plant metabolites [48] and during insect infection by *Photorhabdus luminescens* [49]. Also, the expression regulation of *mdtABC* by the primary regulator BaeR has been observed in vitro in *E. coli* and *Salmonella* as a response to a wide range of stress conditions, including plant metabolites such as tannins and flavonoids [49,50,51]. With this in mind, it would be interesting, in the future, to understand these regulatory molecular mechanisms as well as the chemiotaxis pathways that could serve as a route of dispersion or interaction of the *K. cowanii* Cp1 strain with these biological *Capsicum* seed compounds.

The genome sequencing results of the *K. cowanii* Cp1 strain showed a close phylogenetic relationship with *K. cowanii* Ch1, including the IncF plasmid. Both strains presented virulence genes that are principally related with iron uptake, siderophore, invasion, and motility (Table 1). Interestingly, secretion system component genes were not detected, which is especially relevant because the *K. cowanii* Pa82 strain encodes the type VI secretion system as a virulence factor that causes plant disease [25]. Based on the results observed in the pathogenic test of *K. cowanii* Cp1 in *C. annuum* L. plants and fruits (Figure 2), we can conclude that *K. cowanii* Cp1 is not able to act as a phytopathogen despite the virulence related genes detected. Similar results were observed in *K. cowanii* Ch1 [32], which suggests that both bacterial strains most likely do not represent a potential risk of affecting *Capsicum* plants. However, the fact that the spread of the *Kosakonia* genus could be associated with *Capsicum* seeds is important to consider if the phytopathogenic strains of the *Kosakonia* genus associated with other cultivated plants eventually might cause damage in the *Capsicum* genus through different spread routes. Thus, for instance, *K. cowanii* has been reported as the causal agent of bacterial wilt in tomatoes and patchouli plants and, recently, as an emergent phytopathogen affecting soybean (*Glycine max* Willd) [25,26]. In Eucalyptus trees, symptoms of bacterial blight [27] are presented, and in *Mabea fistulifera* Mart. (Euphorbiaceae), necrotic spots can occur on leaves [28]. Therefore, the monitoring of the microorganism-associated seeds of the *Capsicum* genus in either fresh, dried, or processed products could be relevant in terms of avoiding the bacterial spread that comes with a potential risk of causing infection in susceptible *Capsicum* plants.

On the other hand, it is recognized that the antibiotic resistance in bacteria is part of their natural genetic characteristics and evolution processes, which play important roles during environmental competition [52]. However, it is also one of the greatest emergent problems in human or animal health due to the increase in the prevalence of multidrug-resistant (MDR) bacteria, which have arisen through natural selection due to the abusive overuse of antibiotics in various human activities [52,53]. Therefore, the presence of antibiotic-resistant mechanisms in *K. cowanii* Cp1, such as TEM β-lactamase (which allows for phenotypical resistance to penicillin G, ampicillin, and carbenicillin) is a pressing concern. In addition, the antibiotic efflux pump system that was detected and represented by *acrAB-tolC* and *acrAD-tolC*, respectively, is an intrinsic mechanism of multidrug resistance in the diverse members of Gram-negative bacteria. The antibiotic profile of the pump system includes tetracycline, novobiocin, chloramphenicol, fluoroquinolone, fusidic acid, nalidixic acid, lipophilic antibiotics, and β-lactam antibiotics [54]. Another tripartite efflux system identified was *emrAB-tolC*, which has been demonstrated to induce overexpression in *Escherichia coli*, which causes a resistance to nalidixic acid, thiolactomycin, and nitroxoline [55]. Also, the macrolide transporter *macAB-tolC* has been identified as the most likely cause of erythromycin resistance [56]. The transporter *mdfA/cmr* that has also been identified contributes to multidrug resistance in Gram-negative bacteria [57]. Additionally, the other genes that are linked to the resistance mechanism that was identified—such as transcription factors *marAB*, transcriptional repressor *marR*, target modification, and the protein-altering cell wall charge conferring antibiotic resistance—are shown in Figure 4. All of these resistance mechanisms that were detected in *K. cowanii* Cp1 make it necessary to conduct further work so as to evaluate any potential environmental risks. This is required because of its presence in *Capsicum* seeds and chili powder that are used in human consumption.

The colonization ability of microorganisms depends on the metabolic strategies that are ligated to the environmental factors that promote genetic expression regulation so as to synthetize biomolecules with diverse functions during competition in an ecological niche with a community structure [58]. Thus, for instance, the competition for nutrients could promote a change in growth rate and could generate metabolites with certain effects on their competitors. One class of these compounds are the VOCs, which are a huge variety of molecules that are produced by the different metabolic pathways that have been studied in a diverse array of microorganisms [59]. In this sense, the experimental strategy used in *K. cowanii* Cp1 during a dual experiment with six tested phytopatogenic fungi suggested that the volatile organic compounds produced during the growth of this bacterial strain on TSA medium has inhibitory effects on the mycelial rate growth of *Sclerotinia* sp., *R. solani*, *S. rolfsii*, *A. alternata*, and *C. gloeosporioides*, and this occurs by different mechanisms when compared with *F. oxysporum* (which showed a lower degree of effect). Our evidence, obtained by HS-SPME-GC–MS, demonstrates that the diverse chemical classes of VOCs were produced by *K. cowanii* Cp1 during its growth fermentation on TSA medium (Figure 7). The compounds with a high relative abundance were alcohols (23.5%), which were represented by ethanol (at 5.56%) and 1-butanol-3-methyl (at 4.83%) as the most abundant examples, and ketones (22.97%), where the most abundant was 2-butanone, 3-hydroxy, at 13.52%. In addition, those with a lower relative abundance were the diverse compounds detected from the chemical family of pyrazines, esters, aromatics, acids, hydrocarbons, aldehydes, and aromatics. Interestingly, similar results of VOC production were found in the *K. cowanii* Ch1 strain, where 2-butanone, 3-hydroxy was the most abundant compound (at 10.60%), as well as ethanol (at 5.40%), and 1-butanol-3-methyl (4.88%) [32]. According to diverse evidence, some of these identified compounds may affect plants through a diverse array of mechanisms and through the functionality of fungi cells (thereby causing a disruption of membrane fluidity or wall integrity) as well as alterations in metabolism and redox balance [59]. Although 2-butanone, 3-hydroxy could be one of the principal compounds with antifungal properties in *K. cowanii* Cp1, it is too complicated to understand because it is part of a heterogeneous mix of VOCs that have fluctuating concentrations during the fermentation processes that were tested. This interpretation was confirmed using standard VOCs, where 2-butanone, 3-hydroxy showed an inhibition rate of 82.5% when the highest volume was used (150 μL/plate) compared with acetic acid, benzyl alcohol, or 2-nonanone, which demonstrated high inhibition activities when using a lower volume. In addition, relative peak areas identified were 0.68% for benzyl alcohol, 1.32% for 2-nonanone, and 0.90% for 2-butanone compared with 13.52% for 2-butanone, 3-hydroxy or 5.56% for ethanol (Table 2). Therefore, a mix of VOCs has more impact than individual compounds during biocontrol.

According to the genome sequencing analysis of *K. cowanii* Cp1, metabolic pathways were present that produce 2-butanone, 3-hydroxy through the enzymatic activities of acetolactate decarboxylase and 2,3-butanediol dehydrogenase via the diverse carbon sources that are used in fermentation [60]. Evidence suggests that this molecule, functionally, has significant effects in plants, e.g., during growth development [61], drought tolerance [62], and defense against pathogens [63]. On the one hand, the VOC results suggest that, during fermentation pathways, aldehyde dehydrogenase and alcohol dehydrogenase enzymes could be necessary for ethanol production as well as for the butanal (butyraldehyde) pathway that is required to obtain butanol (which were detected in relatively high abundance in the VOCs from *K. cowanii* Cp1). These alcohol classes are components of the VOCs that are present in a diverse array of microorganisms with antifungal activity [64]. Another interesting compound that was identified as a component of the VOCs was Pyrazine-2,5-dimethyl, with a high relative peak area (6.47%). This compound is produced in diverse bacterial species, and it engages in antifungal activity against *Magnaporthe oryzae*, *Phytophthora capsici*, and *A. solani* [59]. Although other VOCs were produced that had a lower relative-peak abundance (Table 2), these could, nevertheless, be more important, because their antifungal activity has been registered in the mVOC 2.0 Database [65]. Therefore, additional work is necessary to understand the impact of each VOC, on its own or as part of a mixture, on the growth of phytopathogenic fungi strains.

In conclusion, the finding of *K. cowanii* Cp1 as a microorganism that is associated with *Capsicum pubescens* seeds and which has antifungal properties through VOC production opens up the possibility of further exploration of important ecological roles as well as of the plant–bacteria interactions with diverse molecules such as capsaicinoids and phenolic compounds. Additionally, the *Capsicum* genus, cultivated throughout all Mexican territory under different environmental conditions, remains to be explored in order to understand the microbiomes associated with the seeds that are of particular interest in terms of finding potential bacterial strains, such as *Kosakonia cowanii*, for the development of biocontrol agents.

## Figures and Tables

**Table 1:** Virulence-related genes as predicted by VFDB source.

Virulence Factor Classification	Gene Name	Putative Function
Endotoxin	*gtrB*	Bactoprenol glucosyl transferase
Iron uptake, siderophore	*entA*	2,3-dihydro-2,3-dihydroxybenzoate dehydrogenase 2,3-dihydro-2,3-dihydroxybenzoate dehydrogenase of siderophore biosynthesis
	*entB*	Isochorismatase of siderophore biosynthesis
	*fepB*	Ferric enterobactin-binding periplasmic protein FepB
	*fepC*	Ferric enterobactin transport ATP-binding protein FepC
	*fepD*	Ferric enterobactin transport system permease protein FepD
	*fepG*	Ferric enterobactin transport system permease protein FepG
	*entS*	Enterobactin exporter EntS
Secretion system, invasion, motility	*flgC*	Flagellar basal-body rod protein FlgC
	*motA*	Flagellar motor rotation protein MotA
	*flgH*	Flagellar L-ring protein FlgH
	*flgB*	Flagellar basal-body rod protein FlgB
	*flgG*	Flagellar basal-body rod protein FlgG
	*fliG*	Flagellar motor switch protein FliG
	*fliP*	Flagellar biosynthesis protein FliP
	*cheY*	Chemotaxis regulator; transmits chemoreceptor signals to flagellar motor components CheY
	*cheW*	Positive regulator of CheA protein activity (CheW)
	*fliM*	Flagellar motor switch protein FliM
	*fliC*	Flagellin FliC
	*fliA*	RNA polymerase sigma factor for flagellar operon

**Table 2:** The VOCs produced by *K. cowanii* Cp1 and detected by HS-SPME-GC–MS.

Compounds	Retention Time (min)	Relative Peak Area (%)	Chemical Classes	Compounds	Retention Time (min)	Relative Peak Area (%)	Chemical Classes
Carbon dioxide	4.041	1.81	Hydrocarbons	1-Hexanol, 2-ethyl-	39.459	2.22	Alcohols
Ethyl ether	4.466	0.70	Esters				
Methanethiol	4.703	0.42	Alcohols	Benzaldehyde	40.287	0.94	Aldehydes
Acetaldehyde	4.854	0.54	Aldehydes	1-Octanol	42.356	1.62	Alcohols
Acetone	6.176	0.61	Ketones	2-Undecanone	44.335	1.12	Ketones
Butanal	7.610	0.32	Aldehydes	2-Acetylthiazole	45.502	0.54	Other compounds
Ethyl Acetate	8.037	0.29	Esters	Acetophenone	45.741	0.74	Ketones
2-Butanone	8.337	0.90	Ketones	Pyrazine, 2,5-dimethyl-3-(3-methylbutyl)-	46.713	0.55	Pyrazine
Butanal, 3-methyl-	9.088	0.23	Aldehydes	Acetic acid, decyl ester	48.558	0.77	Esters
Ethanol	9.566	5.56	Alcohols	4,4-Dimethyl-2-cyclopenten-1-one	49.412	0.08	Other compounds
2,3-Butanedione	12.247	2.58	Ketones	Cyclodecane	53.105	3.10	Hydrocarbons
Trichloroethylene	12.871	0.21	Other compounds	2-Tridecanone	57.172	1.25	Ketones
Trichloromethane	14.529	0.33	Other compounds	3-Decen-1-ol, acetate, (Z)-	58.325	1.20	Alcohols
Toluene	15.273	0.30	Hydrocarbons	Benzyl alcohol	60.204	0.68	Alcohols
Disulfide, dimethyl	16.91	1.35	Other compounds	Butanoic acid, butyl ester	62.141	4.54	Esters
p-Xylene	21.171	0.12	Other compounds	Propanoic acid, 2-methyl-, 2,2-dimethyl-1-(2-hydroxy-1-methylethyl)propyl ester	63.486	2.12	Esters
1-Butanol	21.744	1.42	Alcohols	Phenylethyl Alcohol	63.944	1.99	Alcohols
Ethanol, 2-methoxy-	23.279	0.95	Alcohols	Ethanol, 2,2′-oxybis-	68.575	0.51	Alcohols
2-Heptanone	24.063	0.26	Ketones	Cyclododecane	71.619	4.29	Hydro-carbons
1,3-Diazine	24.981	0.48	Other compounds	2-Propenal, 3-phenyl-	73.169	0.28	Aldehydes
1-Butanol, 3-methyl-	25.259	4.83	Alcohols	2-Nonadecanone	74.724	0.58	Ketones
Pyrazine, methyl-	27.947	1.15	Pyrazine	Octanoic Acid	76.216	0.68	Acids
2-Butanone, 3-hydroxy-	29.244	13.52	Ketones	Triacetin	76.667	0.84	Other compounds
2-Propanone, 1-hydroxy-	29.817	0.09	Ketones	2,4,7,9-Tetramethyl-5-decyn-4,7-diol	78.388	1.90	Alcohols
Pyrazine, 2,5-dimethyl-	30.931	6.47	Pyrazine	Benzoic acid, 4-tert-butyl-, 3,5-dichloro-4-pyridyl ester	79.388	0.19	Esters
Pyrazine, 2,6-dimethyl-	31.245	0.27	Pyrazine	Benzoic acid, 2-amino-, methyl ester	83.037	0.12	Esters
1-Hexanol	32.875	0.20	Alcohols				
Dimethyl trisulfide	33.789	0.20	Other compounds	Methyl 8-methyl-nonanoate	84.445	0.51	Esters
Pyrazine, 2-ethyl-6-methyl-	34.218	0.12	Pyrazine	Hexanedioic acid, dibutyl ester	85.423	0.44	Esters
Pyrazine, 2-ethyl-5-methyl-	34.484	0.43	Pyrazine	Dodecanoic acid, 3-hydroxy-	85.693	1.13	Acids
2-Nonanone	34.997	1.32	Ketones	Diethyl phthalate	88.452	0.39	Other compounds
Pyrazine, 2-methyl-5-(1-methylethyl)-	35.597	0.14	Pyrazine	Indole	90.108	0.11	Aromatics
Pyrazine, 3-ethyl-2,5-dimethyl-	37.062	1.30	Pyrazine	Phthalic acid, hex-2-yn-4-yl isobutyl ester	95.053	1.10	Other compounds
Octanoic acid, ethyl ester	37.407	0.43	Esters				
Acetic acid, octyl ester	39.183	0.21	Esters				

## Data Availability

The data presented in this work are available from the corresponding authors upon request.

## Supplementary Materials

The following supporting information can be downloaded at: https://www.mdpi.com/article/10.3390/microorganisms11102491/s1, Figure S1. Dual-culture assay on PDA. *K. cowanii* Cp1 and *S. rolfsii* were confronted. Mycelial and bacterial growth was register during 2 days of growth at 28 °C. Figure S2. Antibiotic resistance profile of *K. cowanii* CP1 strain
