# Supplementary material for: Volatile Organic Compounds Produced by Kosakonia cowanii Cp1 Isolated from the Seeds of Capsicum pubescens R & P Possess Antifungal Activity
Source: Microorganisms. 2023 Oct 4;11(10):2491. doi: 10.3390/microorganisms11102491 (PMC10609226; doi:10.3390/microorganisms11102491)
Supplement: Supplementary file 1 [file microorganisms-11-02491-s001.zip › microorganisms-2653889-supplementary.pdf]

Supplementary Figures

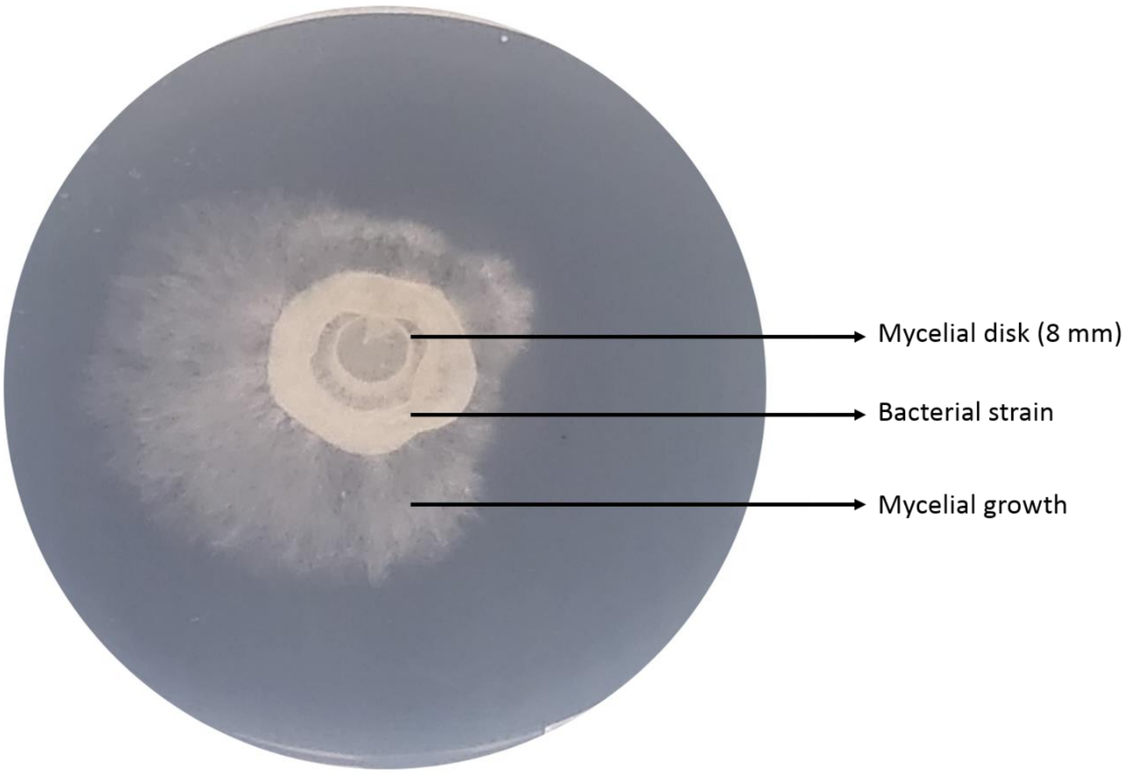

Figure S1. Dual-culture assay on PDA. *K. cowanii* Cp1 and *S. rolfsii* were confronted. Mycelial and bacterial growth was register during 2 days of growth at 28 °C.

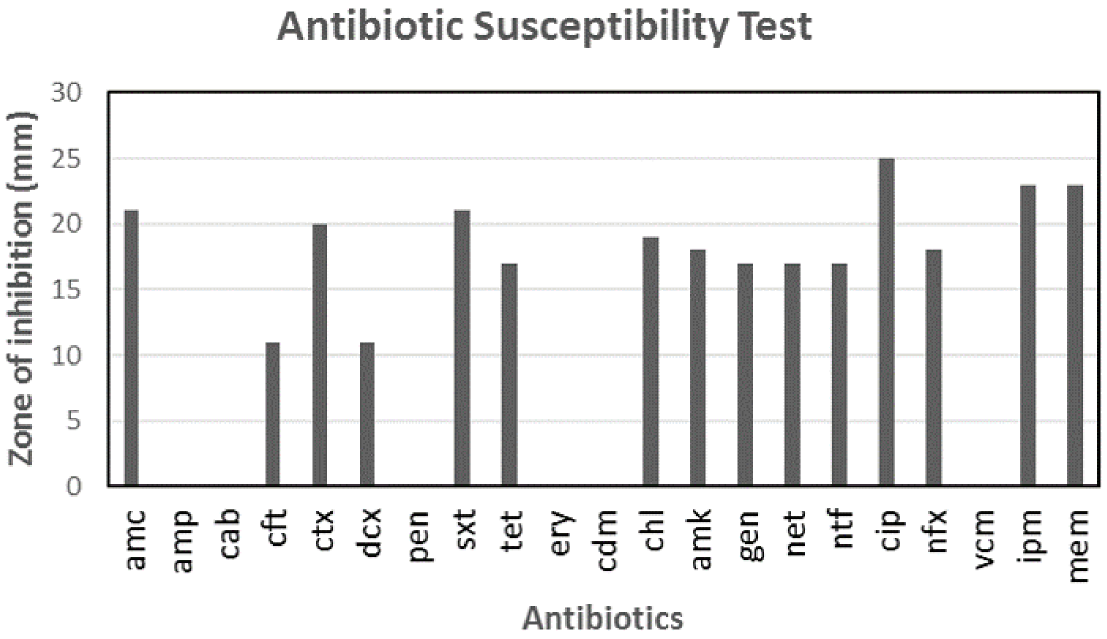

Figure S2. Antibiotic resistance profile of *K. cowanii* CP1 strain. Antibiotic discs (Oxoid) with amikacin (amk 30 µg), ampicillin (amp 10 µg), amoxi-cillin/clavulanic acid (amc 20/10 µg), carbenicillin (cab 100 µg), cefalotin (cft 30 µg), cefotaxime (ctx 30 µg), chloramphenicol (chl 30 µg), ciprofloxacin (cip 5 µg), clindamycin (cdm 30 µg), dicloxacillin (dcx 1 µg), erythromycin (ery15 µg), gentamicin (gen 10 µg), imipenem (ipm 10 µg), meropenem (mem 10 µg), netilmicin (net 30 µg), nitrofurantoin (ntf 300 µg), norfloxacin (nfx 10 µg), penicillin (pen10 U), tetracycline (tet 30 µg), trime-thoprim-sulfamethoxazole (sxt 25 µg) and vancomycin (vcm 30 µg) were used for suscep-tibility testing.
